# Biodistribution of the Bombesin Receptor-Targeted Radiopharmaceutical Precursor BBN/C1-C2 in a Prostate Cancer Model

**DOI:** 10.17691/stm2025.17.6.03

**Published:** 2025-12-29

**Authors:** E.A. Beloborodov, E.V. Iurova, D.R. Dolgova, A.A. Ermakova, D.E. Sugak, A.N. Fomin, Y.V. Saenko

**Affiliations:** Researcher, Laboratory of Research and Development of Peptide Drugs and Vaccines, S.P. Kapitsa Technological Research Institute; Ulyanovsk State University, 42 Leo Tolstoy St., Ulyanovsk, Russia, 432017; Junior Researcher, Laboratory of Research and Development of Peptide Drugs and Vaccines, S.P. Kapitsa Technological Research Institute; Ulyanovsk State University, 42 Leo Tolstoy St., Ulyanovsk, Russia, 432017; PhD, Senior Researcher, Laboratory of Molecular and Cellular Biology, S.P. Kapitsa National Research Institute of Technology; Director of the Research Medical and Biological Center; Associate Professor, Department of Physiology and Pathophysiology; Ulyanovsk State University, 42 Leo Tolstoy St., Ulyanovsk, Russia, 432017; Research Assistant, Laboratory of Research and Development of Peptide Drugs and Vaccines, S.P. Kapitsa Technological Research Institute; Ulyanovsk State University, 42 Leo Tolstoy St., Ulyanovsk, Russia, 432017; Junior Researcher, Laboratory of Research and Development of Peptide Drugs and Vaccines, S.P. Kapitsa Technological Research Institute; Ulyanovsk State University, 42 Leo Tolstoy St., Ulyanovsk, Russia, 432017; PhD, Senior Researcher, Laboratory of Research and Development of Peptide Drugs and Vaccines, S.P. Kapitsa Technological Research Institute; Ulyanovsk State University, 42 Leo Tolstoy St., Ulyanovsk, Russia, 432017; DSc, Leading Researcher, Laboratory of Research and Development of Peptide Drugs and Vaccines, S.P. Kapitsa Technological Research Institute; Ulyanovsk State University, 42 Leo Tolstoy St., Ulyanovsk, Russia, 432017

**Keywords:** bombesin, knottin, targeted therapy, peptide toxin, bombesin receptor

## Abstract

Gastrin-releasing peptide receptor (GRPR) is a G protein-coupled receptor expressed in the central nervous system, the gastrointestinal tract, the pancreas, and the adrenal cortical tissue regulating their physiological functions. In addition to normal tissues, GRPR is overexpressed in many solid cancers. At present, several radiolabeled ligands targeting GRPR have been introduced into clinical practice for cancer diagnostics and radioligand therapy. However, there were found the high uptake of radiopharmaceuticals in normal organs and low bioavailability of the drugs caused by their stability. Therefore, GRPR radioantagonists with improved proteolytic stability and longer residence time in tumor foci with low uptake in non-target organs are currently being sought to improve the therapeutic efficacy.

**The aim of the study** was to analyze the biodistribution of the BBN/C1-C2 molecule created on the basis of bombesin and knottin and its accumulation dynamics in the tumor in an *in vivo* model.

**Materials and Methods:**

The study analyzed the biodistribution of Cy7.5-labeled BBN/C1-C2 peptide based on bombesin and knottin U5-Sth1a and produced using solid-phase peptide synthesis. The investigation was carried out on a mouse model Nu/Nu with a prostate cancer solid tumor (PC-3 culture) transplanted into the right side, expressing GRPR, using real-time surface fluorescent imaging on days 2 and 5 after intravenous administration of the molecules under study.

**Results:**

The biodistribution analysis showed the selective binding of the BBN/C1-C2 molecule to a tumor, as well as its holding power on the tumor surface for up to 5 days without internalization into cells with low accumulation in normal organs and tissues.

**Conclusion:**

We managed to obtain a stable molecule; however, U5-Sth1a toxin tropic to ion channels was used as a scaffold, so the resulting molecule retained the domain responsible for attaching to the target channel and typical for knottin. In the future, when using the molecule as a ligand for radiotherapy, it can have a negative effect on the patient’s heart due to the supplementary radiation load. In this regard, the BBN/C1-C2 molecule requires the extension study.

## Introduction

Gastrin-releasing peptide receptor (GRPR) is a G protein-coupled receptor expressed in the central nervous system, the gastrointestinal tract, the pancreas, and the adrenal cortical tissue, regulating their physiological functions [1]. In addition to normal tissues, GRPR is overexpressed in many solid cancers. For instance, GRPR is associated with the growth of prostate carcinoma [2], mammary adenocarcinoma [3], and colorectal cancer [4]. Therefore, the development of GRPR-targeted pharmaceuticals is a long-range objective for mass lesion imaging and therapy.

Currently, there have been introduced into clinical practice several radiolabeled ligands targeting GRPR and based on a gastrin-releasing peptide (GRP) analog isolated from amphibian — bombesin (BBN) [5–8]. However, the patients and preclinical animal models were found to have high doses of radiopharmaceuticals accumulated in normal organs, particularly in the pancreas [7, 8]. In addition to the high concentration of radiopharmaceuticals in non-target organs, there is one more significant problem complicating cancer therapy — pharmaceutical stability. A bombesin molecule shows good accumulation in a tumor, however, it has low proteolytic stability [9]. In an attempt to stabilize bombesin, for example, by using a motif [DPhe6,Leu13-NHEt] (demobisin), the molecule affinity for GRPR and the pharmaceutical accumulation by a tumor appear to improve, however, low proteolytic stability still persists [10]. One more bombesin modification, N4-AMA-DIG-[DPhe6,Sar11,LeuNHEt13]BBN, has demonstrated its high resistance to proteolysis [11]; it determined current active studies being carried out on patients. In this regard, to enhance the therapeutic efficacy, it is of current interest to search for GRPR radioantagonists with improved proteolytic stability and the longer residence time in tumor foci with low uptake in non-target organs.

Our previous study used knottin from spider venom as a stabilizing molecule by positioning a short bombesin peptide in the domain between the first and the second cysteine residue (BBN/C1-C2) [12]. Having proved the chemical and radiochemical stability of the obtained molecule, we analyzed the BBN/C1-C2 molecule ability to bind to the cancer cell surface. The present study involved an *in vivo* imaging technique to show the biodistribution of the created molecule and its accumulation dynamics in a tumor. For comparison we used GRP peptide and a native toxin, on the base of which BBN/C1-C2 was developed.

## Materials and Methods

### Peptide synthesis and quality control

The peptides were synthesized on an automated peptide synthesizer ResPep SL (Intavis, Germany) on a solid resin (TentaGel; Intavis, Germany) based on Fmoc-chemistry. As an activator we used HBTU (Chemical Line, Russia) dissolved in dimethyl formamide (DMF) (Chemical Line, Russia). Deprotection was performed by 4-methyl piperidine (PanReac Applichem, Spain) in DMF, capping — using 5% acetic anhydride (Acros Organics, Belgium). After synthesis the peptides were removed from the resin using the solution containing 95% trifluoroacetic acid (Acros Organics, Belgium), 5% triisopropylsilane (PanReac Applichem, Spain), and 5% deionized water.

After synthesis the chemical purity of the peptides was analyzed by reversed-phase chromatography; we used high-efficiency liquid chromatography: LC-20AD XR (Shimadzu, Japan) equipped with a spectrophotometric detector and a column Luna C18(2) (Dr. Maisch, Germany). The elution was gradient, its mobile phase consisted of deionized water and acetonitrile. The acetonitrile content in a mobile phase was increased from 5% at the beginning of the study to 100% at the end of the study within 40 min. The flow rate was 1 ml/min. Detection was performed at a wavelength of 215 nm, using mass spectrometry with the MALDI-TOF MS FLEX mass spectrometer (Bruker Daltonics, Germany).

The peptides were purified using the chromatograph AutoPure25 (Inscinstech, China) by reversed-phase chromatography using the column Galaksil EF-C18H (GALAK Chromatography Technology Co., Ltd, China). The elution was gradient, its mobile phase consisted of deionized water and acetonitrile. The acetonitrile content in a mobile phase was increased from 5% at the beginning of the study to 100% at the end of the study within 90 min. The flow rate was 1 ml/min. Detecting was at wavelength 215 nm.

After purification the toxin and BBN/C1-C2 were exposed to folding in the solution containing 10 mM reduced and 1 mM oxidized glutathione in 0.1 М Tris-HCl (PanEco, Russia) at pH 8.0. The peptides were incubated in the solution at 4°С for 24 h with constant stirring.

### Fluorescent dye-labeling of peptides

For imaging the *in vivo* biodistribution of peptides, we used Cyanine7.5 (hereinafter Cy7.5) (LLC “Lumiprobe RUS”, Russia), which provides the fluorescence in near infrared band (Ex/Em — 788/808 nm) ensuring the dye to fluoresce in the region, where nature molecules fail to fluoresce. Labeling was made using Cy7.5 NHS ester to bind the dye to the peptide amino group according to a standard manufacturer’s protocol. For this purpose, the peptides were dissolved in deionized water at the concentration of 1 mg/ml, then the solution was mixed with 1 M sodium bicarbonate to achieve pH 8.3 of the resulting reaction mixture. After that the solution was added the dye dissolved in DMSO, in 8-fold excess molar quantity in relation to the peptide. The mixture was incubated for 3 h, at room temperature, constantly stirring. Three hours later, the mixture was embedded in 10-fold excess of icy ethyl alcohol and centrifuged at 21,000 g, 4°С for 30 min. After that the removal of the unbound dye was repeated.

### Stability analysis of peptide complexes

We studied the stability of both — the whole structures (Cy7.5-BBN/C1-C2, Cy7.5-GRP, Cy7.5-U5-Sth1a) and also the peptide complexes with Cy7.5. So, all peptides were incubated in the phosphate-buffered saline (pH 7.4) for 144 h, at 37°С. Every 24 h we drew the samples and analyzed them using high-efficiency liquid chromatography LC-20AD XR (Shimadzu, Japan), as described above. The stability of the complex with the dye was fixed similarly to the peptide stability fixation, as following from the accumulation of the free dye in samples.

### In vivo fluorescent imaging of a tumor

The study was carried out on Nu/Nu line male mice aged 9–10 weeks, weighing 26–28 g.

The work with animals was approved by the committee of the Institute of Medicine, Ecology, and Physical Education, Ulyanovsk State University. Before the experiment started, the Bioethics Committee assessed the study design and considered the protocol to correspond to international standards of law and ethics to work with laboratory animals. The procedures were performed in accordance with the European Convention for the Protection of Vertebrate Animals used for Experimental and other Scientific Purposes (Strasburg, 2006).

Since Cy7.5-GRP peptide stability was limited, firstly, 48 h at 37°С in phosphate-buffered saline, there were two time points used in the study — 2 and 5 days. Two weeks prior to the investigation, the animals were subcutaneously inoculated into the right side with PC-3 cells (1 mil cells in RPMI-1640, 100 μl (PanEco, Russia), and matrigel, 100 μl (ABW, China)) to form a tumor. When the tumor reached its optimal size (150–200 mm3), the animals (n=10) were divided into the following groups: group 1 animals were administered a pure dye (n=1); group 2 — were given Cy7.5-GRP peptide (n=3); group 3 — were administered Cy7.5-U5-Sth1a toxin (n=3); group 4 — had Cy7.5-BBN/C1-C2 administered (n=3). The animals were anesthetized with 3% isoflurane inhalation (Chemical Iberica, Spain) at the rate of 1 L/min within 10 min, then the dose was reduced up to 2%. The peptides were injected into the caudal vein at the rate of 19 nmol/kg. The imaging included ventrodorsal view in a dorsal positioning by the system VISQUE InVivo Smart-LF (Vieworks, Korea) using light-emitting diodes (LED) on filters (Ex/ Em — 740–790/810–860 nm), exposure — 1000 ms, binning — 2×2. The findings were processed using the software CleVue (Vieworks, Korea). On day 5 the animals were sacrificed using cervical dislocation; their main organs being harvested (the liver, the stomach, the lungs, the heart, the pancreas, the spleen, the small intestine, and the kidneys) to assess the biodistribution and the tumor. The fluorescent signal from the organs was studied in the program CleVue (Vieworks, Korea). The regions of interest were manually determined along the organ borderline; then using the software, we calculated the total fluorescence for each region. Finally, the average fluorescence of the peptides in the organ was calculated, it being normalized to the organ weight [13, 14].

### Statistical processing of data

The findings were processed in OriginPro (v. b9.5.0.193; OriginLab Corporation, USA), where the statistical difference was calculated using Mann–Whitney test. Skewness and kurtosis normality test was used to determine the distribution character. Bonferroni adjustment was applied to eliminate the effect of multiple comparisons. The differences were considered significant if p<0.01.

## Results

**Figure 1.**
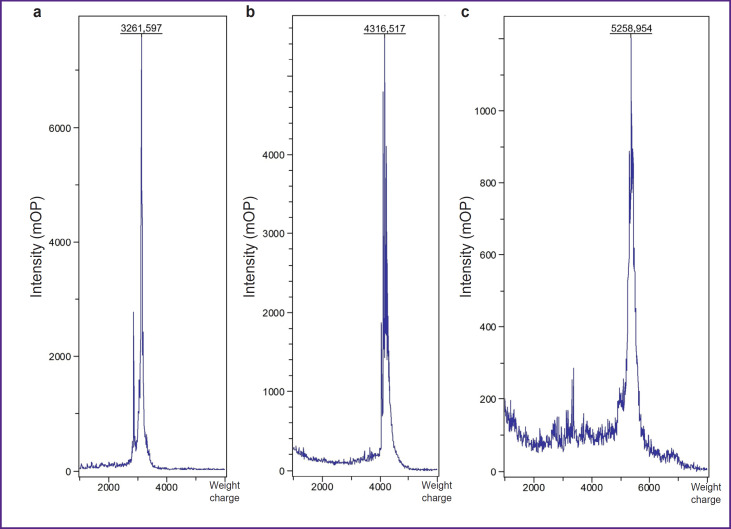
Mass-spectrograms of GRP (a), U5-Sth1a (b) and BBN/C1-C2 (c)

There were obtained 3 peptides with purity over 90%: GRP — a short peptide with affinity to GRPR receptors; U5-Sth1a toxin, which was used as a scaffold for bombesin stabilization; and BBN/C1-C2 developed on the basis of U5-Sth1a bombesin and knottin (Figure 1).

**Figure 2.**
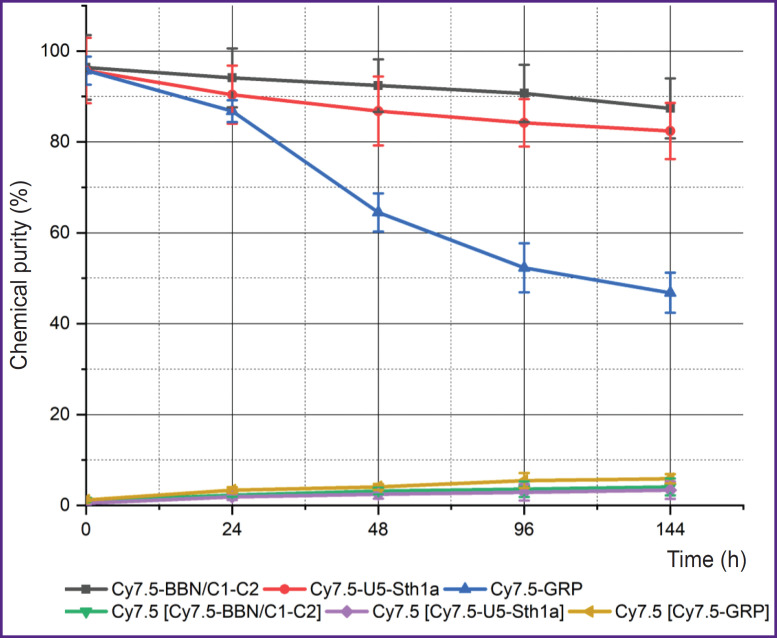
Chemical stability of Cy7.5-GRP, Cy7.5-BBN/C1-C2, and Cy7.5-U5-Sth1a peptides and the complexes of Cy7.5-peptides when incubated in phosphate-buffer saline (pH 7.4) at 37°С The free dye percentage in the sample is indicated when being measured

After synthesis the peptides were successfully labeled with a fluorescent Cy7.5 probe. There were analyzed the stability of both: the whole molecules and also the [Cy7.5]-peptide complexes (Figure 2). Cy7.5-GRP peptide stability was found to significantly decrease during the first 48 h (from 95.7 to 64.5%) and keep decreasing within the following 144 h reaching 46.8%. Cy7.5-BBN/C1-C2 and Cy7.5-U5Stha1 peptides were more stable in phosphate-buffered saline when heated. The chemical purity of Cy7.5-BBN/C1-C2 decreased from 96.4 to 87.4% in 144 h, while Cy7.5-U5Stha1 — from 95.7 to 82.4% in 144 h. The stability analysis of the complexes of peptides and the dye showed 5% dye to be lost when being incubated in phosphate-buffered saline in all groups.

**Figure 3.**
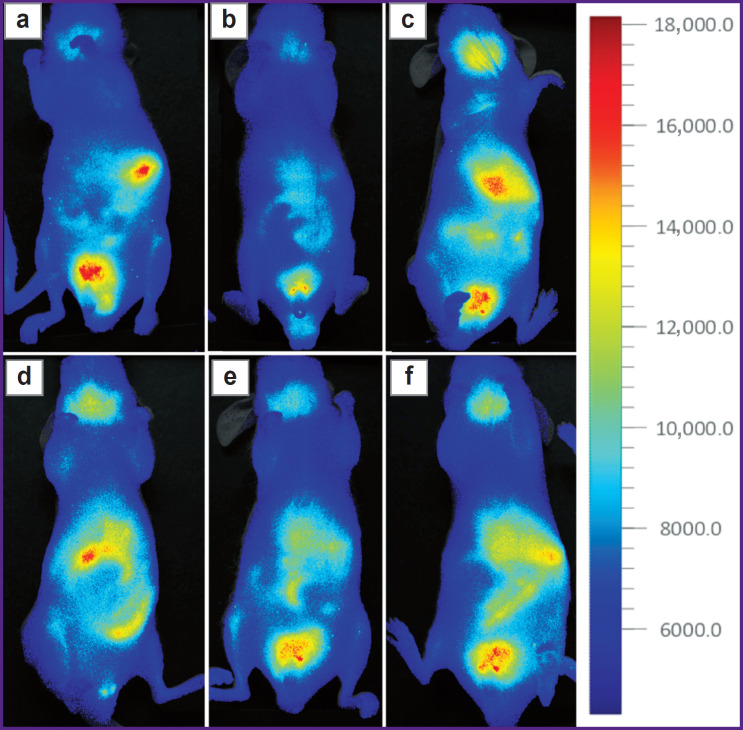
Distribution of Cy7.5-GRP (a, d), Cy7.5-U5-Sth1a (b, e) and Cy7.5-BBN/C1-C2 (c, f) in the view of the organs in Nu/Nu mice in 2 days (a–c) and 5 days (d–f) after the intravenous injection at a dose of 19 nmol/kg

The imaging of the distribution of three molecular structures in organs (Figure 3) showed the main region of GRP binding on day 2 was the spleen, and the organ to excrete — the kidney. It was due to significantly smaller size of the short peptide in relation to U5-Sth1a and BBN/C1-C2. For the first 2 days there was the BBN/ C1-C2 accumulation in the liver and spleen region, as well as in the bladder and the submandibular salivary glands (an enhanced signal in the bladder view from all three molecules can be related to insignificant residues of the free dye). On day 5 an increased signal for GRP was detected in the view of GIT organs and the pancreas (the foodstuff residue signal was recorded in the small intestine view). U5-Sth1a and BBR/C1-C2 also kept excreting by the bladder, however, the signal in the view of the stomach, the pancreas, the spleen, and the liver was still enhanced.

**Figure 4.**
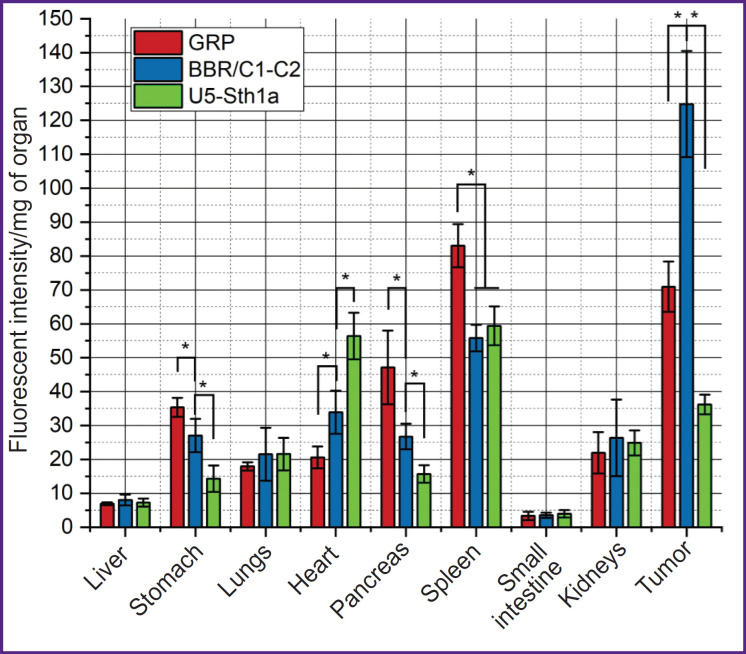
Semiquantitative distribution analysis of GRP, BBR/C1-C2, and U5-Sth1a at a dose of 19 nmol/kg in the main organs on day 5 after their administration The semi-quantitative analysis is based on fluorescent intensity normalized with the organ weight. The error bars indicate standard deviation; * p<0.01

The biodistribution analysis on day 5 after peptide intravenous administration demonstrated the following (Figure 4). The main organs were analyzed. The fluorescent signal intensity of an organ was integrated, decreased with the background noise, and normalized to the organ weight. The main organ of accumulating all three peptides was the spleen. Along with the spleen, the short GRP peptide was localized in the stomach and the pancreas. BBN/C1-C2 signal detected in these organs was significantly weaker, although it was higher than the U5-Sth1a toxin signal. It should be particularly noted that the fluorescent signal from the U5-Sth1 toxin and the BBN/C1-C2 peptide was increased in the heart (although to a lesser extent) that is likely to be related to the nature target of the chosen toxin. The signal for all three molecules in the rest organs was detected.

**Figure 5.**
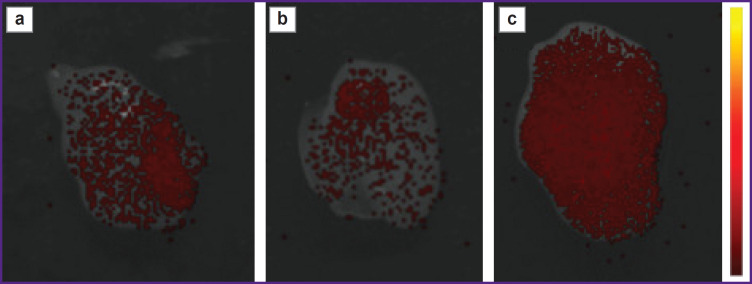
The tumor imaging when administering Cy7.5-GRP (a), Cy7.5-U5-Sth1a (b) and Cy7.5-BBN/C1-C2 (c) on day 5

The localization of labeled peptides in the tumor on days 2 and 5 was considered separately (Figure 5). It can be observed that on day 2 GRP was detected in the tumor to a greater extent than BBN/C1-C2; however, on day 5 the situation changed. The BBN/C1-C2 signal appeared to significantly exceed the GRP signal.

## Discussion

The peptides of bombesin family act as growth factors for tumors of different types. Bombesin and its analog in mammals — GRP — have structural and functional similarity. Both peptides perform the functions of gastrointestinal hormones, neurotransmitters, and autocrine growth factors for tumor cells [15]. Since GRPR receptors overexpress in different tumors, GRP and BBN are intensively studied as theranostic ligands targeting tumor cells. For example, there was demonstrated the significant accumulation of peptides in the cells of PC-3, DU-145, MDA-MB-231, T-47-D, HeLa lines [16, 17].

The key feature of these peptides is their ability to internalize inside the cells, acting as receptor agonists. At early stages of developing pharmaceuticals, this ability was an important condition for high and long-term absorption of a tumor by a pharmaceutical [18, 19]. However, currently, it appears to be a substantial deficiency, since radiolabeled agonists provide high absorption of radionuclides. One more major drawback of the ligands based on GRPR agonists is their absorption by normal non-tumor cells, particularly, the pancreatic cells. So, the potent GRPR agonist — AMBA (DOTA-Gly-4-aminobenzoyl-BBN(7–14)) — demonstrates excellent results in imaging and therapy in the models with the prostate cancer and breast cancer xenotransplants [20, 21], that makes it one of effective bombesin analogs, which passed a wide spectrum of clinical trials. Despite this, the patients with AMBA therapy were found to have significant absorption of the pharmaceutical by the pancreas and the gastrointestinal tract [22]. In addition, the phase I of the escalation study carried out in patients with metastatic castrate-resistant prostate cancer was terminated due to the severe side effects caused by the GRPR activation following the injection of therapeutic doses [23].

Stimulating effects of bombesin agonists in several cancer types in a human have moved forward to developing GRPR antagonists as anti-tumor drugs able to bind to a receptor with high affinity without its activation. Extensive research [24–26] of the relation of the structure and biological activity have resulted in the rise of various GRPR antagonists obtained by modifying the peptide backbone of native motifs BBN or GRP. Into the category of such modifications can be referred the truncation of the ultimate C-terminal methionine, alkylamidation, esterification of the open carboxyl group of the penultimate residue, the reduction of peptide bonds, as well as the replacement of key amino acids by their D-analogs or other residues. It promoted the rise of a variety of potent GRPR antagonists demonstrating the antiproliferative activity in mammal cells expressing GRPR and also in tumors in mice [17].

The next development stage reveals one more critical problem of peptide ligands targeting GRPR. Low proteolytic stability serves a key factor in developing a pharmaceutical regardless of its biological function as an agonist or antagonist. The modifications related to the agonist modification into an antagonist can partly solve the problem, but insufficiently [8, 27–30]. In the present study, as a stabilizing molecule, we used knottin — a toxin, which is able to form a cystine node stabilized by several disulfide bonds due to the presence of several cysteine residues. A bombesin molecule (BBN) was placed between the first and the second cysteine in order to enhance stability, since the distinctive feature of knottins is their proteolytic stability. However, when incorporating into the relatively large toxin molecule, bombesin loses its ability to internalize inside a cell. Our previous study [12] showed BBN/C1-C2 to have the increased chemical stability preserving the ability to bind to GRPR of the cell surface without internalization into cells. The present study analyzed the *in vivo* biodistribution of BBN/C1-C2, and the ability to bind to tumor cells. The investigation was carried out comparing BBN/C1-C2 with a short GRP peptide and U5-Sth1a toxin, on the base of which there was created the BBN/C1-C2 molecule.

The GRPR-targeted GRP peptide location zone was shown to be the stomach and the pancreas (see Figures 3 and 4). The BBR/C1-C2 peptide was also observed in the mentioned organs, although to a lesser extent. The analysis of fluorescent intensity dynamics in the tumor is the additional confirmation of BBN/C1-C2 targeting. On day 5 after administration there was recorded the higher BBN/C1-C2 signal in relation to GRP (see Figure 5). However, there comes up a problem associated with using knottin as a scaffold for bombesin stabilization. The toxins from arthropods venom (U5-Sth1a from *Scytodes thoracica* spider) have certain biological target. Primarily, such target is limited by a certain ion channel (voltage-dependent calcium, sodium, potassium, etc. channels). When interacting with a target channel, the toxins cause either its closure or modulate its conductivity. Finally, such interaction results in changing the membrane potential and ion concentration in cells. In the present case, a biological target for U5-Sth1a toxin is unknown. However, by parallel studies we could clarify that calcium voltage-dependent channels are likely to be a toxin target, and the toxin itself acts as a blocker [31]. Since the location of voltage-dependent calcium channels is also the myocardium (as well as the smooth myocytes in vascular walls and any hollow organs, such as the stomach, the intestine, the bladder, etc.) [32], we can observe that both the toxin (see Figure 4) and also BBN/C1-C2 attach in the heart, although BBN/C1-C2 attaches to a far lesser extent.

## Conclusion

We managed to obtain a stable molecule containing bombesin enclosed in the knottin backbone, able for a long time to hold on the surface of a tumor, which expresses GRPR without internalization inside the cells. However, as far as U5-Sth1a toxin tropic to ion channels was used as a scaffold, so the resulting molecule retained the domain responsible for attaching to the target channel and typical for knottin. In the future, when using the molecule as a ligand for radiotherapy, it can increase the radiation load on the patient’s heart. In this regard, the BBN/C1-C2 molecule requires the extension study.
